# Diagnosis of Tuberculous Meningitis: Integrating Clinical Assessment and Molecular Diagnostics

**DOI:** 10.3390/diagnostics16040552

**Published:** 2026-02-13

**Authors:** Jorge E. Leiva-Ordoñez, Beatriz Quintero

**Affiliations:** School of Medicine, Universidad Técnica Particular de Loja, Loja 110101, Ecuador; jeleiva1@utpl.edu.ec

**Keywords:** tuberculosis, meningeal, cerebrospinal fluid, molecular diagnostic techniques, nucleic acid amplification techniques, high-throughput nucleotide sequencing

## Abstract

Tuberculous meningitis is the most severe form of tuberculosis and remains associated with high mortality and substantial neurological disability, particularly among children and people living with HIV. Early diagnosis is challenging because of nonspecific clinical manifestations, the limited discriminatory value of cerebrospinal fluid cytochemical analysis, and the low sensitivity of conventional microbiological methods. This narrative review synthesizes contemporary evidence on the diagnostic approach to tuberculous meningitis, integrating clinical assessment, paraclinical cerebrospinal fluid findings, conventional microbiology, and molecular diagnostic tools. Clinical scoring systems, including the uniform case definition (Lancet consensus score), improve diagnostic stratification but do not replace microbiological confirmation. Molecular assays have transformed diagnostic pathways by enabling rapid detection of *Mycobacterium tuberculosis*, although their performance is influenced by bacillary burden, cerebrospinal fluid volume, HIV status, and disease stage. Complementary molecular techniques and advanced sequencing approaches provide additional diagnostic value in selected paucibacillary cases or when first-line tests are negative. Integrated diagnostic algorithms that combine clinical evaluation with stepwise molecular testing improve diagnostic accuracy and support earlier treatment initiation. Ongoing challenges include limited access to molecular platforms, variability in laboratory capacity, and the need for standardized multimodal diagnostic pathways applicable across diverse healthcare settings.

## 1. Introduction

Tuberculosis (TB) remains one of the leading causes of mortality due to a single infectious agent. In 2023, an estimated 10.8 million cases were reported worldwide, with the highest burden in Southeast Asia (44%) and Africa (25%), followed by the Western Pacific (18%), the Eastern Mediterranean (8.2%), the Americas (2.9%), and Europe (2.5%). Seven countries account for nearly two thirds of the global incidence: India, Indonesia, China, the Philippines, Pakistan, Nigeria, and South Africa [1,2,3]. Although *Mycobacterium tuberculosis* primarily causes pulmonary disease, extrapulmonary forms represent approximately 15–20% of cases [4].

Tuberculous meningitis (TBM) accounts for 1–5% of global tuberculosis cases and represents the most lethal manifestation among extrapulmonary forms. This condition is associated with severe neurological sequelae and long-term disability, which underscores its clinical relevance and the need for timely diagnosis [2,5,6,7]. In adults, an estimated 64,000 cases occur annually, with an overall mortality of 27% and rates reaching up to 70% in sub-Saharan Africa [8,9]. In 2019, approximately 24,000 children developed TBM and 11,000 died, corresponding to an estimated case fatality rate of 67% [10]. Among people living with HIV, the risk of tuberculosis is 15–20 times higher than in the general population, while TBM-related mortality in this group ranges from 38% to 57% [11,12]. In addition, the high frequency of neurological complications—including hydrocephalus (50–75%), hyponatremia (52%), tuberculomas (27–51%), cerebral infarctions (14–16%), and persistent sequelae in children—further amplifies the clinical burden of TBM [5,13,14].

The central challenge therefore lies in achieving an early and reliable diagnosis. However, cerebrospinal fluid (CSF) in TBM is typically paucibacillary, and the available volume is often insufficient to maximize the diagnostic yield of laboratory tests [15,16,17]. Although culture retains a confirmatory role, its sensitivity remains limited (50–70%), and results require several weeks [5,18,19]. Molecular techniques, such as Xpert MTB/RIF and Xpert Ultra, have improved diagnostic turnaround time, but their performance continues to be influenced by bacillary burden, the volume of cerebrospinal fluid processed, and operational constraints in resource-limited settings [12,19,20]. In this context, it is essential to review the utility of available diagnostic strategies, including clinical, microbiological, and molecular approaches, in order to provide a clear overview of their advantages, limitations, and future perspectives within a stepwise diagnostic framework for TBM.

## 2. Conventional Diagnosis of Tuberculous Meningitis

### 2.1. Initial Clinical Assessment and Diagnostic Challenges

The diagnosis of TBM poses a major clinical and microbiological challenge, driven by the nonspecific clinical presentation, the paucibacillary nature of CSF, and the absence of a fully validated international reference standard for diagnostic confirmation. Within this context, the diagnostic process variably integrates clinical criteria, radiological findings, CSF cytochemical evaluation, and conventional microbiological tests, to which molecular assays have been added more recently. Consequently, the set of criteria applied in both clinical practice and research exhibits substantial variability across regions and levels of care where studies are conducted [21,22,23].

To date, no single, fully standardized international clinical guideline exists for the diagnosis of TBM, which has led to the development of partial recommendations issued by different international scientific bodies. Key documents include the British guidelines on central nervous system tuberculosis published by the British Infection Society in 2009, the most recent recommendations from the IDSA (2023), and the consolidated diagnostic modules of the WHO (Module 3: Diagnosis, 2025). Although these guidelines do not focus exclusively on TBM and differ in their degree of standardization, they consistently highlight the diagnostic complexity imposed by the low bacillary burden in CSF and the need to integrate clinical, microbiological, and molecular domains to improve diagnostic accuracy [22,23,24,25].

From a historical perspective, the British guideline proposed an integrative diagnostic model grounded in clinical–biochemical correlation and supported by neuroimaging as the cornerstone of the initial approach [24]. Subsequently, the IDSA recommendations incorporated the concept of Composite Reference Standard (CRS) as a methodological framework for validating diagnostic tests in extrapulmonary TB, acknowledging the limitations of culture as the sole reference standard [22]. The CRS integrates microbiological evidence, clinical and radiological findings, and, in selected cases, treatment response, thereby allowing classification of cases into hierarchical categories of diagnostic probability, even in the absence of bacteriological confirmation [26].

In parallel, the WHO demonstrated a shift toward molecular diagnosis by recommending automated nucleic acid amplification tests (NAAT) on CSF as the initial diagnostic tool in patients with suspected TBM. Within this framework, the WHO identified Xpert MTB/RIF Ultra as the best-performing assay, with a sensitivity of approximately 88% and a specificity of around 96%, displacing smear microscopy and culture as initial tests, while retaining culture as a confirmatory method because of its value for establishing antimicrobial susceptibility profiles of *M. tuberculosis* [25]. Taken together, these recommendations reveal a progressive transition from clinico-empirical models to a microbiological–molecular paradigm, with emphasis on comprehensive CSF analysis and the combined validation of diagnostic tools [22,24,25].

### 2.2. Clinical Features, Neuroimaging, and Uniform Case Definition in Tuberculous Meningitis

In this context, the need to standardize diagnostic criteria led to the development of the uniform case definition proposed by Marais et al., internationally known as the Lancet consensus score, which integrates clinical, radiological, and microbiological domains within a structured diagnostic classification system (Table 1 and Table 2) [27]. Although initially conceived as a research tool, this definition has become the reference framework against which diagnostic tests for TBM are currently evaluated, as it contextualizes their performance across different clinical categories and levels of diagnostic probability [2].

The Lancet consensus score classifies patients with suspected TBM into four categories: definite, probable, possible, and non-tuberculous meningitis. The definite category requires identification of *M. tuberculosis* in CSF by smear microscopy, culture, or a validated molecular assay. The probable and possible categories are determined through a scoring system that integrates clinical criteria, CSF cytochemical findings, and neuroimaging results (Table 2) [27]. Within the clinical domain, the Lancet consensus score incorporates clinical symptoms that are nonspecific and frequently overlap with other forms of meningitis [27]. From an epidemiological perspective, a history of contact with an active TB case within the preceding 12 months is frequently reported in children (>50%), although this variable does not contribute to the score calculation [27].

Neuroimaging represents a central component of the Lancet consensus score, as it supports clinical suspicion and allows assessment of disease extent and complications. The main radiological criteria included are hydrocephalus, basal meningeal enhancement, tuberculomas, and cerebral infarctions [27]. The simultaneous presence of basal enhancement, cerebral infarction, and hydrocephalus achieves a specificity of 100%, whereas basal meningeal enhancement is the most sensitive finding, with a reported sensitivity of 89% [27].

Importantly, the frequency of neuroimaging abnormalities varies according to the imaging modality used and the clinical characteristics of the population studied [28]. For example, a pediatric study identified differences in the prevalence of key TBM-related findings depending on the imaging technique. Computed tomography showed a higher frequency of hydrocephalus compared with magnetic resonance imaging (55% vs. 36%), whereas both modalities revealed similar rates of basal meningeal enhancement (29% vs. 26%, respectively) [28]. Comparative studies indicate that magnetic resonance imaging generally outperforms computed tomography for detecting brainstem lesions, tuberculomas, meningeal enhancement, hydrocephalus, and lacunar infarctions. In addition, magnetic resonance imaging facilitates monitoring of treatment response, positioning it as the imaging modality with the best overall performance for neuroanatomical evaluation in TBM [2,29].

The diagnostic value of neuroimaging extends to specific clinical scenarios. In patients with markedly elevated CSF protein levels or spinal symptoms, contrast-enhanced spinal magnetic resonance imaging becomes particularly relevant due to the high frequency of spinal meningeal involvement described in this subgroup [30]. In this context, Ma et al. [29] developed a radiomics-based model using T2 and FLAIR sequences to identify alterations not visible in the basal cisterns, although this approach requires clinical validation in larger cohorts before broader application. Overall, neuroimaging findings constitute a cornerstone of the Lancet consensus score; however, they are insufficient on their own to establish a definitive etiological diagnosis [27].

Although the Lancet consensus score has become the most widely used methodological framework in TBM research, its performance may vary according to epidemiological profile, local TB burden, and availability of diagnostic tools [2]. Available studies show more consistent behavior when comparing diagnostic test performance in the “probable TBM” category, with greater overlap observed among cases classified as “possible TBM” [2]. At this stage, critical evaluation of alternative clinical models becomes relevant, as their performance is influenced by derivation cohort characteristics and their applicability to heterogeneous populations remains limited.

### 2.3. Alternative Clinical Models for Differential Diagnosis

The Thwaites’ Diagnostic Score, developed in 2002 to differentiate TBM from acute bacterial meningitis, uses simple clinical variables and the proportion of neutrophils in CSF (Table 1 and Table 2) [21]. The discriminatory accuracy of the Thwaites’ Diagnostic Score was evaluated in a multicenter study including 395 adults with subacute and chronic meningitis, which found that the score differentiated acute bacterial etiology from other causes in 99% of patients [31]. Therefore, while useful for distinguishing acute bacterial meningitis, the Thwaites’ score shows limited ability to guide specific etiological assessment of TBM, a limitation that has driven the development of more refined clinical models [31].

Subsequently, several clinical models have been proposed to improve discrimination between tuberculous and bacterial meningitis, generally combining routinely available clinical and cerebrospinal fluid parameters (Table 1 and Table 2) [32,33,34]. Although these models report high diagnostic performance, they differ in complexity, variable selection, and feasibility, reflecting attempts to balance accuracy with clinical applicability in diverse settings. Among these approaches, a recently proposed model appears particularly relevant from a clinical perspective, as it relies on a limited number of routinely available clinical, laboratory, and radiologic variables organized within a structured risk-stratification scheme. The model showed excellent discriminatory performance and outperformed commonly used tools, including the Lancet Consensus Scoring System [35]. Importantly, its development was accompanied by a web-based calculator that allows clinicians to estimate the probability of TBM in real time, classify patients into risk categories, and obtain suggested clinical actions, facilitating practical bedside use. However, external validation in independent cohorts is still required before this approach can be broadly implemented in routine clinical practice [35].

In an approach aimed at differentiating TBM from viral meningitis, Lee et al. [36] developed a clinically oriented score based on six routine clinical and cerebrospinal fluid variables, showing good diagnostic performance and practical bedside applicability. This model is particularly useful in early decision-making when clinical overlap between TBM and viral meningitis complicates initial assessment (Table 1 and Table 2) [35]. Broader diagnostic approaches have subsequently incorporated differentiation across multiple meningitis etiologies (Table 1 and Table 2). Lu et al. [30] developed a comprehensive multivariable model integrating clinical, cerebrospinal fluid, and neuroimaging data to distinguish TBM from viral, bacterial, and cryptococcal meningitis, highlighting the role of advanced imaging in complex cases. In contrast, Liu et al. [37] proposed a more pragmatic nomogram based on routinely available clinical, cerebrospinal fluid, and limited neuroimaging variables to differentiate TBM from bacterial and viral meningitis, improving clinical feasibility in high-burden settings.

In summary, while alternative clinical models for the differential diagnosis of tuberculous meningitis generally show encouraging diagnostic performance, meaningful comparison across studies is challenged by marked variability in study design, patient populations, and validation strategies (Table 1 and Table 2) [21,30,31,32,33,34,35,36,37]. As a result, their usefulness in routine practice largely depends on the clinical context, local epidemiology, and access to diagnostic resources. In this setting, these models are best understood as aids to early clinical reasoning rather than standalone diagnostic tools, with definitive etiological confirmation and treatment decisions still relying on microbiological and molecular evidence.

**Table 1:** Clinical scoring systems and predictive models for the differential diagnosis of tuberculous meningitis.

Model	Intended Use	Cut-Offs/Categories	Key Advantages and Limitations	Development/ Validation Limitations	Best-Use Settings
Marais et al. (2010) [27] (Lancet consensus score)	Uniform case definition for diagnostic standardization in research and clinical reporting.	**Definite:** microbiological confirmation. **Probable:** ≥12 points (with neuroimaging available) or ≥10 points (without neuroimaging). **Possible:** 6–11 points (with neuroimaging) or 6–9 points (without neuroimaging). **Not TBM:** <6 points.	**Advantages:** Widely adopted; integrates clinical features, CSF, neuroimaging, and evidence of extrapulmonary TB. **Limitations:** Limited usefulness for immediate bedside decision-making.	**Country:** multinational. **Model type:** case definition. **Sample size:** NA. **Performance** **(AuROC):** NA. **External validation:** NA.	Clinical research, epidemiology, and comparative diagnostic studies.
Thwaites’ Diagnostic Score (Sulaiman et al. 2020 [31])	Initial triage. Differential diagnosis: TBM vs. acute bacterial meningitis (ABM).	≤4 points suggests TBM. >4 points suggests BM.	**Advantages:** Simple bedside application. **Limitations:** Lower performance in subacute/chronic meningitis; poor discrimination in PLWH and children; does not incorporate molecular testing.	**Country:** multinational. **Model type:** clinical score. **Sample size:** 395 (162 TBM). **Performance** **(AuROC):** <0.5. **External** **validation:** Yes (multicenter revalidation).	Resource-limited settings; early triage.
Yang et al. (2020) [32]	Differential diagnosis: TBM vs. BM.	TDI: ≥0 suggests TBM.	**Advantages:** Better discrimination than traditional scores; easy to apply. **Limitations:** Not evaluated in PLWH; single-country cohort; no external validation.	**Country:** China. **Model type:** clinical score. **Sample size:** 103 (58 TBM). **Performance** **(AuROC):** NR. **External** **validation:** No.	Tertiary-care centers.
Luo et al. (2021) [33]	Differential diagnosis: TBM vs. bacterial meningitis (BM).	Cut-off ≈ 0.54 suggests TBM.	**Advantages:** Incorporates an immunological biomarker; high performance with external validation. **Limitations:** Requires T-SPOT; the study did not include non-bacterial meningitis etiologies.	**Country:** China. **Model type:** logistic model. **Sample size:** 279 (115 TBM/164 BM). **Performance** **(AuROC):** 0.949 (derivation)/0.923 (external validation). **External** **validation:** Yes (independent cohort).	Centers with access to immunology testing.
Wen et al. (2022) [34]	Differential diagnosis: TBM vs. BM.	Clinical score (TDI): TDI ≥ 7 classifies as TBM. TDI < 7 classifies as BM.	**Advantages:** User-friendly model; strong internal performance. **Limitations:** Limited external validation; retrospective study.	**Country:** China. **Model type:** clinical score. **Sample size:** 380 (114 TBM). **Performance** **(AuROC):** 0.954. **External** **validation:** Yes (limited).	Referral hospitals
Krongsut et al. (2025) [35]	Multivariable clinical prediction model (web-based calculator available) Differential diagnosis: TBM vs. BM.	Provides an estimated probability of TBM with low, intermediate, and high-risk categories	**Advantages:** Very high discriminatory performance. Web-based calculator for real-time risk stratification and bedside clinical decision support. **Limitations:** Single-center study; lacks external validation.	**Country:** Thailand. **Model type:** web-based model. **Sample size:** 377 (142 TBM). **Performance** **(AuROC):** 0.978. **External** **validation:** NA.	Hospitals in TB-endemic areas; resource-limited settings.
Lee et al. (2018) [36]	Differential diagnosis: TBM vs. VM.	≥4 points suggest TBM.	**Advantages:** Good performance; feasible bedside use. **Limitations:** Restricted to TBM vs. VM; no external validation.	**Country:** South Korea. **Model type:** clinical score. **Sample size:** NA. **Performance** **(AuROC):** 0.901. **External validation:** No.	General hospitals.
Lu et al. (2021) [30]	Differential diagnosis: TBM vs. BM, VM, and cryptococcal meningitis.	≥5 points suggest TBM.	**Advantages:** Integrates clinical data, CSF, and neuroimaging; prospective validation. **Limitations:** Requires MRI; not evaluated in PLWH; lacks international validation.	**Country:** China. **Model type:** nomogram. **Sample size:** 382 (113 TBM). **Performance** **(AuROC):** 0.923 (derivation)/0.884 (prospective validation). **External** **validation**: Yes.	Tertiary-care centers with access to neuroimaging.
Liu et al. (2023) [37]	Differential diagnosis: TBM vs. BM and VM.	Probabilistic nomogram: Approx. score 22–24 indicates intermediate probability (~50%). Approx. score 27–30 indicates high probability (~80–90%).	**Advantages:** Excellent discrimination (AuROC > 0.95); integrates clinical and neuroimaging predictors. **Limitations:** Does not incorporate molecular diagnostics; validated within one country.	**Country:** China. **Model type:** nomogram. **Sample size:** 322 (102 TBM). **Performance** **(AuROC):** 0.962. **External** **validation:** No.	High TB-burden settings.

Notes: This table summarizes the main clinical scoring systems proposed for the differential diagnosis of TBM. Scores should be interpreted as decision-support tools and integrated with cerebrospinal fluid findings, neuroimaging, and microbiological/molecular testing; a negative score does not exclude TBM. Performance metrics (when reported) reflect the study setting and reference standard used and may not be directly comparable across cohorts. Models are not presented in chronological order; instead, they are organized according to their practical applicability, ease of use, and overall clinical utility across different settings. TBM, tuberculous meningitis; ABM, acute bacterial meningitis; BM, bacterial meningitis; VM, viral meningitis; CSF, cerebrospinal fluid; PLWH, people living with HIV; AUC/AUROC, area under the receiver operating characteristic curve; MRI, magnetic resonance imaging; NA, not available; NR, not reported [21,30,31,32,33,34,35,36,37].

**Table 2:** Comparison of alternative clinical and clinic–paraclinical diagnostic models for tuberculous meningitis.

Author, Year of Publication	Marais, 2010 [27]	Thwaites’ Score, 2002 [21]	Yang, 2020 [32]	Luo, 2021 [33]	Wen, 2022 [34]	Krongsut, 2025 [35]	Lee, 2018 [36]	Lu, 2021 [30]	Liu, 2023 [37]
**Study characteristics**									
Sample Size (TBM events)	NA	395 (162)	103 (58)	279 (115)	380 (114)	377 (142)	NA	382 (113)	322 (102)
**Key Predictors Included**									
cough ≥ 2 weeks	−	−	+	−	−	−	−	−	−
disease duration	+	+	+	−	+	+	−	+	+
impaired consciousness	+		−	−	−	+	+	−	+
Weight loss	−	−	−	−	−	+	−	−	+
cerebral infarction (imaging)	+	−	−	−	−	+	−	+	+
Serum sodium	−	−	+	−	−	−	+	+	−
CSF neutrophil percentage	−	+	+	−	−	−	−	+	−
CSF lymphocyte percentage	+	+	−	+	−	+	−	−	+
CSF glucose	+	−	−	−	−	+	−	−	+
CSF proteins	+	−	−	−	+	+	+	−	−
CSF chloride	−	−	−	−	+	−	−	−	−
immunological biomarkers	−	−	−	+	−	−	−	−	−
chest X-ray suggestive of TB	+	−	−	−	−	−	−	−	−
evidence of extrapulmonary TB	+	−	−	−	−	−	−	+	−

Notes: This table summarizes alternative clinical and clinic–paraclinical diagnostic models developed to differentiate tuberculous meningitis from other causes of meningitis. In addition to the variables included in each model, the table reports key study characteristics, including country or study setting, sample size, and the number of tuberculous meningitis events, providing context on the populations in which these models were derived. Most models rely on routinely available clinical variables and cerebrospinal fluid parameters, which enhance their feasibility in clinical practice. However, the majority were derived from retrospective, single-center cohorts and lack external validation in independent populations. Furthermore, advanced molecular diagnostic assays were not systematically incorporated into these models. Therefore, their diagnostic performance should be interpreted with caution, and these tools should be regarded as complementary rather than substitutes for microbiological and molecular confirmation. “+” indicates inclusion in the model; “−” indicates exclusion; TBM, tuberculous meningitis; TB, tuberculosis; CSF, cerebrospinal fluid; NA, not available [21,30,31,32,33,34,35,36,37].

### 2.4. Cytochemical Determinants and Biomarkers in CSF

CSF analysis continues to play a central role in the early diagnostic evaluation of tuberculous meningitis, as it offers rapid and widely accessible information that helps frame initial clinical suspicion. The characteristic CSF profile, typically showing lymphocytic pleocytosis, elevated protein levels, and reduced glucose concentration, reflects a subacute inflammatory process. However, these abnormalities are inherently nonspecific and often overlap with other infectious causes of meningitis, which limits their ability to define tuberculous etiology on their own (Table 3) [2,27]. Recent comparative analyses support CSF lactate as the most robust cytochemical parameter among routine markers, particularly in definite TBM, while confirming the limited specificity of glucose, protein concentration, and pleocytosis across different meningitis etiologies [38]. For this reason, routine cytochemical parameters are best interpreted as supportive findings rather than definitive diagnostic criteria.

Among classical CSF biomarkers, adenosine deaminase (ADA) has received the greatest attention in the literature. Nevertheless, its practical value is constrained by marked variability across studies and a pronounced dependence on host immune status. Elevated ADA levels may also be observed in bacterial meningitis and other inflammatory conditions, particularly among people living with HIV, thereby reducing its specificity and limiting its usefulness as a stand-alone diagnostic marker (Table 3) [14,39,40,41,42]. Other routinely available CSF biomarkers, including lactate, C-reactive protein, procalcitonin, ferritin, and lipoarabinomannan (LAM), may contribute additional contextual information during the diagnostic workup. However, none of these markers provide sufficient specificity to independently confirm TBM. Their main clinical contribution lies in supporting decision-making, especially in resource-limited settings, rather than in establishing definitive etiological diagnoses (Table 3) [16,42,43,44]. Accordingly, these biomarkers are best viewed as adjunctive tools that help contextualize clinical suspicion rather than as definitive diagnostic tests.

Beyond classical enzymatic and cytochemical markers, increasing attention has been directed toward host-response immunological biomarkers, including interferon-γ-related pathways and angiogenic factors, although current evidence remains largely exploratory and cohort-specific [42,45]. Among these, vascular endothelial growth factor A (VEGF-A) has emerged as one of the most consistently reported candidates, particularly when incorporated into multi-marker panels, where it appears to enhance diagnostic performance in selected populations, including children (Table 3) [16]. Despite these promising findings, the lack of assay standardization, absence of validated cut-off values, and limited external validation currently restrict its translation into routine clinical practice. Overall, CSF biomarkers should be interpreted within an integrated diagnostic framework that combines clinical assessment, neuroimaging, and microbiological or molecular testing, rather than being used in isolation.

**Table 3:** Cerebrospinal fluid cytochemical analysis and biomarkers for the diagnosis of tuberculous meningitis.

Biomarker	Typical Findings in TBM	Cut-Offs	Clinical Value	Main Limitations	References
Cytochemical parameters					
CSF glucose	Decreased (<2.2 mmol/L or CSF/serum ratio < 40–50%)	<30–40 mg/dL (depending on study and population)	Supports initial diagnostic suspicion. Moderate sensitivity and variable specificity.	Low specificity. Overlaps with other meningitis etiologies. May be normal in early TBM or in PLWH. Inferior diagnostic performance compared with CSF lactate.	[38,46]
CSF Lactate	Markedly elevated, especially in definite TBM	>5.5 mmol/L	Supports initial diagnostic suspicion (strong biomarker). Metabolic biomarker with high sensitivity and good specificity for definite TBM. Useful as a rapid and accessible test, including point-of-care methods.	Not specific for TBM. Also elevated in bacterial meningitis, cryptococcal meningitis, cerebral ischemia, seizures, and subarachnoid hemorrhage. Lower performance in probable/possible TBM.	[16,38,43]
CSF proteins	Elevated (>1 g/L, frequently 1–3 g/L)	>1 g/L (Supports diagnostic orientation)	Supports diagnosis in subacute/chronic meningitis. Reinforces suspicion of TBM versus viral meningitis. Associated with higher risk of complications (hydrocephalus, vasculitis)	Elevation is not exclusive. Increased in other etiologies such as fungal meningitis, bacterial meningitis, meningeal carcinomatosis, and neurosarcoidosis.	[27,46]
Cytochemical parameters					
Lymphocytic pleocytosis	10–500 cells/µL; >50% lymphocytes	>10 cells/µL with lymphocytic predominance (supports diagnostic orientation)	Supports diagnosis in subacute/chronic meningitis.	Overlap with viral and fungal meningitis. May be neutrophilic or mild in early TBM or in PLWH. Low standalone discriminative power.	[27,46]
Erythrocyte count	Generally low. May be mildly elevated in complicated TBM	>6.5 erythrocytes/µL (cohort- specific)	Auxiliary finding/indicator of complexity. May reflect meningeal vascular involvement.	Poor discriminative utility. Low specificity; influenced by traumatic lumbar puncture and other causes of bleeding. No universal cut-off.	[46]
Enzymatic biomarkers					
ADA	Higher CSF ADA activity in TBM compared with non-TB meningitis (relative increase).	5–10 U/L (cut-offs used across different studies; no standardized threshold).	Complementary enzymatic biomarker Good overall performance. Accessible and available.	No universal cut-off; performance influenced by immune status (e.g., PLWH) and coinfections.	[39,40,41,42]
Immunological biomarkers					
FN-γ	Higher CSF IFN-γ levels compared with non-TB meningitis (relative increase)	Not standardized (SMD 0.45–1.73 across etiological comparisons).	Complementary use. Good discriminatory performance versus cryptococcal, viral, and bacterial meningitis; supports inclusion in multimarker diagnostic panels.	No validated cut-off; high inter-study heterogeneity and assay-dependent variability.	[42,45]
VEGF-A	Higher VEGF-A concentrations in CSF and plasma compared with non-TB meningitis (relative increase).	Not standardized.	Complementary use. Moderately consistent evidence; improves diagnostic performance when incorporated into multimarker models, particularly in combination with inflammatory cytokines.	No validated clinical cut-off; evidence derived mainly from selected cohorts and exploratory analyses; limited external validation.	[42]
TB Ag/PHA ratio (derived from T-SPOT)	Higher TB antigen to phytohaemagglutinin (TB Ag/PHA) ratio in TBM compared with bacterial meningitis (relative increase).	Not standardized.	Complementary use. Supports differential diagnosis between TBM and bacterial meningitis in adults when IGRA-based testing is available.	Requires availability of IGRA (T-SPOT). Not specifically validated for TBM. Limited utility in PLWH due to impaired T-cell responses.	[42]
Immunological biomarkers					
VEGF-A + IFN-γ ± MPO	Concurrent relative increase in multiple host-response biomarkers, reflecting combined inflammatory and vascular activation.	Not defined.	Complementary use. Improves diagnostic discrimination between TBM and bacterial meningitis when incorporated into multivariable models, as reflected by increased AUC.	Limited external validation; lack of standardized analytical platforms and reproducible cut-off values restrict clinical implementation.	[42]
Pathogen-derived markers					
LAM (FujiLAM > AlereLAM)	Positive in TBM, particularly in people living with HIV	Qualitative	Complementary rule-in test High specificity. Useful as a confirmatory assay in PLWH.	Low and variable sensitivity. Limited performance in HIV-negative individuals.	[16,44]

Note: This table summarizes the CSF cytochemical parameters and biomarkers evaluated to support the diagnosis of TBM. Biomarkers are organized according to their clinical role and the maturity of the supporting evidence, rather than their chronological development. First, routinely available CSF cytochemical parameters (glucose, lactate, proteins, pleocytosis, and red blood cell count) are presented. These tests are widely accessible and play a central role in structuring the initial diagnostic suspicion, particularly in subacute and chronic meningitis, although their diagnostic specificity is limited and substantial overlap exists with other infectious and non-infectious meningitis etiologies. Second, the enzymatic biomarker ADA is included. Although extensively studied and readily available, ADA lacks a universal cut-off value, and its diagnostic performance is influenced by immunological context, including HIV infection and concomitant coinfections. Consequently, ADA should be interpreted as a complementary biomarker rather than a stand-alone diagnostic test. Finally, immunological biomarkers (e.g., IFN-γ, VEGF-A) and multimarker panels are grouped together. These biomarkers demonstrate discriminative potential in selected cohorts and may improve diagnostic performance when incorporated into multivariable models; however, their clinical applicability remains constrained by limited standardization, assay-dependent variability, absence of reproducible cut-off values, and scarce external validation. Pathogen-derived biomarkers (e.g., LAM) are presented as complementary confirmatory rule-in tools due to their high specificity, particularly in people living with HIV, despite variable sensitivity. Reported cut-off values should be interpreted as indicative rather than definitive, as they depend on the study population, analytical method, and reference standard employed. Overall, these biomarkers should be integrated with clinical assessment, neuroimaging, and microbiological or molecular testing, rather than used in isolation. ADA, adenosine deaminase; IFN-γ, interferon gamma; VEGF-A, vascular endothelial growth factor A; MPO, myeloperoxidase; LAM, lipoarabinomannan; IGRA, interferon-gamma release assay; TB Ag/PHA ratio, *M. tuberculosis*-specific antigen to phytohaemagglutinin ratio derived from IGRA; HIV, human immunodeficiency virus; PLWH, people living with HIV; TBM, tuberculous meningitis; SMD, Standardized Mean Difference.

### 2.5. Conventional Microbiological Methods: Scope and Limitations

Etiological confirmation of TBM relies on the direct identification of *M. tuberculosis* by culture. This method remains the reference standard against which molecular assays are evaluated, as it enables definitive confirmation and antimicrobial susceptibility testing [2,16]. However, the marked paucibacillary nature of CSF in TBM limits the performance of these methods and sustains a relevant gap between clinical suspicion and microbiological confirmation [47].

Smear microscopy, although accessible and widely used in resource-limited settings, exhibits very low sensitivity. Clinical series have reported positivity rates ranging from 15% to 40% when large CSF volumes are processed and multiple smears are performed, with wide variability depending on processing methods [2,40]. A meta-analysis estimated an overall smear sensitivity of 8% and a specificity close to 100%, indicating that more than 90% of cases would be missed if this technique were used alone [48]. Consistently, a comparative study using the uniform case definition as reference reported a smear sensitivity of only 10% in definite cases, albeit with 100% specificity [49]. Taken together, smear microscopy retains an operational role but is inadequate as a standalone method for etiological confirmation in suspected TBM.

Mycobacterial culture constitutes the main confirmatory tool and the benchmark for validation of new diagnostic assays. Its performance depends largely on CSF volume and processing methods. For culture, guidelines recommend using at least 6 mL of CSF, centrifugation at 3000× *g* for 20 min, and inoculation of the cellular sediment to increase bacillary recovery [2,16,40]. Under optimal conditions, culture sensitivity ranges from 50% to 60% [40], whereas operational settings have reported negativity rates varying between 15% and 75% [16].

Two main culture categories are used for *M. tuberculosis*: solid and liquid media. Solid media, such as Löwenstein–Jensen, Ogawa, and Middlebrook 7H10/7H11, require 4–8 weeks to demonstrate microbial growth and show variable sensitivity, with reports as low as 4.3% [16,50]. In contrast, automated liquid systems, such as BACTEC MGIT 960, reduce time to detection (10–18 days) and provide higher sensitivity (>10%), although they show greater susceptibility to cross-contamination compared with solid media [19,40]. Overall, both solid and liquid media demonstrate high specificity for TB; therefore, a positive culture confirms the diagnosis. Nevertheless, in TBM, culture yield from CSF remains constrained by low bacillary burden [16]. A unique advantage of culture lies in its ability to isolate the organism for subsequent phenotypic and genotypic antimicrobial susceptibility testing, as well as molecular surveillance of resistant strains [19,40].

**Figure 1:**
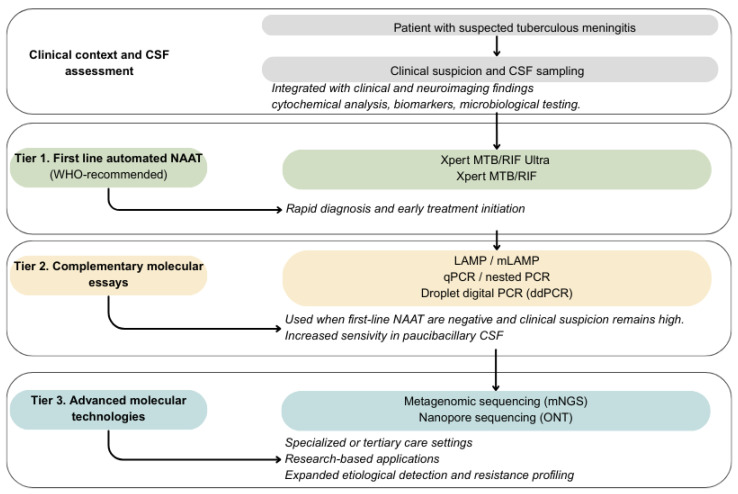
Integrated overview of the diagnostic pathway for TBM.

In summary, conventional microbiological methods remain essential for etiological confirmation and resistance profiling in TBM. However, limited sensitivity and prolonged time to results restrict their utility as isolated tools for initial clinical decision-making. These limitations have driven the adoption of molecular amplification techniques, which are addressed in the following section. An integrated overview of the diagnostic pathway for TBM is presented in Figure 1.

This figure illustrates a conceptual, stepwise diagnostic framework for tuberculous meningitis, integrating clinical suspicion, cerebrospinal fluid (CSF) assessment (including cytochemical analysis, biomarkers, and microbiological testing), and molecular diagnostics. First-line automated nucleic acid amplification tests (NAAT), recommended by international guidelines, are positioned as programmatic diagnostic tools to support rapid diagnosis and early treatment initiation. When first-line NAAT are negative and clinical suspicion remains high, complementary molecular assays may be employed to improve diagnostic sensitivity in paucibacillary CSF. Advanced molecular technologies, such as metagenomic next-generation sequencing and nanopore sequencing, are depicted as tertiary or research-based tools, reserved for specialized centers when the etiological diagnosis remains uncertain or when expanded pathogen detection and resistance profiling are required. Overall, the figure emphasizes that diagnostic decision-making in tuberculous meningitis relies on the integrated interpretation of clinical, paraclinical, microbiological, and molecular data, rather than on any single test in isolation.

## 3. Molecular Diagnosis: Emerging Technologies, Clinical Application, and Perspectives

### 3.1. First-Line Molecular Assays (Automated NAAT)

Automated NAAT have progressively replaced traditional microbiological methods because of their faster turnaround time, high degree of automation, and superior ability to detect *M. tuberculosis* in paucibacillary samples (Table 4). According to recommendations from the WHO and IDSA, Xpert MTB/RIF and Xpert MTB/RIF Ultra constitute first-line tests for the initial diagnosis of TBM in adults, pediatric populations, and people living with HIV [2,22].

Xpert MTB/RIF is an automated cartridge-based PCR assay that enables detection of *M. tuberculosis* and mutations associated with rifampicin resistance within approximately two hours, with consistently high specificity (≥98%) across most clinical studies [40]. In contrast, its sensitivity shows marked dependence on the reference standard used, the diagnostic category considered, and the volume of CSF processed, ranging from 13% to 100% (Table 4).

The highest sensitivity was observed when culture was used as the reference standard, both in adults (30–100%) [51,52,53,54,55] and in children (93%) [56], as well as in adults when the Lancet consensus score was applied to definite cases (75–100%) [57,58,59]. A meta-analysis including more than 4000 CSF samples further confirmed substantial variability in sensitivity according to the reference standard, with higher values against culture (63–71%) than against a composite reference standard (51–61%) [51].

Overall, although the sensitivity of Xpert MTB/RIF is influenced by multiple preanalytical and methodological factors, the assay maintains consistently high specificity and performs better in scenarios with higher bacillary burden. Its diagnostic utility increases when combined with biomarkers or clinical–molecular models, which improve sensitivity without compromising specificity [55,60].

Xpert MTB/RIF Ultra represents an evolution of the Xpert system, with improved diagnostic performance derived from an expanded reaction chamber and the incorporation of multicopy targets (IS6110/IS1081), thereby reducing the limit of detection and increasing sensitivity in paucibacillary settings (Table 4). Ultra has demonstrated sensitivities of 64–81% in definite cases and 35–52% in probable cases, while maintaining specificities between 91% and 100%, depending on the reference standard applied [58,61,62,63]. Although results vary across studies, most comparisons show higher sensitivity for Xpert MTB/RIF Ultra than for Xpert MTB/RIF, at the expense of relatively lower specificity. A meta-analysis including more than 600 CSF samples reported that Ultra was more sensitive than Xpert (64% vs. 37%), without demonstrating superiority in specificity [61]. This increased sensitivity has also been replicated when definite TBM [56,58], a composite reference standard [62,64], or culture [56] were used as reference standards.

In contrast, specificity results for Ultra have been heterogeneous. Some studies reported 100% specificity for both assays when the Lancet consensus score [58] or CRS [64] served as the reference, whereas others observed lower specificity for Ultra than for Xpert when CRS [62] or culture [56] was used. In summary, the available evidence indicates that the overall diagnostic accuracy of Xpert MTB/RIF Ultra exceeds that of Xpert MTB/RIF across both microbiological and composite reference standards, despite differences in relative specificity between the two assays [56,58,61,62,64]. The lower specificity reported for Ultra may reflect variations in bacillary burden, CSF processing, and methodological heterogeneity, rather than a true increase in false-positive results, consistent with its enhanced analytical capacity to detect very low bacillary loads or residual nucleic acid fragments not recovered by conventional culture [56,62].

### 3.2. Complementary and Emerging Molecular Assays

A range of complementary molecular assays has been developed to increase diagnostic sensitivity or expand detection when first-line NAAT yield negative results. These technologies rely on different amplification principles and vary in technical complexity, required sample volume, and clinical applicability. The main alternative and emerging platforms evaluated for TBM diagnosis are summarized in Table 4.

Loop-mediated isothermal amplification (LAMP) enables DNA replication at 60–65 °C using a strand-displacing polymerase and a set of primers targeting multiple regions of the sequence of interest. This architecture generates large quantities of amplicons within less than one hour and shows high tolerance to inhibitors, with visual or fluorescent readouts that do not require thermocyclers, favoring use in resource-limited settings [55,60]. However, complex primer design increases the risk of nonspecific amplification and amplicon contamination, which necessitates strict standardization and limits multiplexing [65,66]. In TBM studies, a monogenic TB-LAMP targeting IS6110 achieved a sensitivity of 53% and specificity of 78% [67]. A multitarget LAMP version (MLAMP) demonstrated substantially superior performance (Sens: ≥88%; Spec: 100%) against both CRS and culture, outperforming even Xpert MTB/RIF Ultra in paucibacillary samples [63]. This improvement was attributed to simultaneous targeting of multicopy (IS6110, IS1081) and single-copy (*sdaA*) genes.

Quantitative real-time PCR (qPCR) platforms use fluorescence-based amplification detection, enabling relative quantification of *M. tuberculosis* DNA. This technique requires thermocyclers, standardized extraction procedures, and specific reagents, but provides high specificity and flexibility in target selection [47,68,69]. TrueNat MTB/MTB Plus, a portable qPCR assay targeting the nrdB gene, showed a sensitivity of 83.75% and specificity of 88.57% compared with Xpert MTB/RIF, although the WHO considers current evidence insufficient to recommend its use in CSF [25,70]. Alerta MTB qPCR achieved sensitivities close to 81% in people living with HIV, but lacks specificity data and is therefore recommended only within sequential diagnostic algorithms [47]. CapitalBio PCR reported a sensitivity of 48% and specificity of 100% against culture using 2 mL of CSF, positioning it as a confirmatory assay with high positive predictive value [69]. Overall, qPCR maintains excellent specificity but variable sensitivity, constrained by molecular targets and CSF volume, and serves a complementary role in stepwise algorithms when Xpert MTB/RIF or Ultra yield negative results.

Droplet digital PCR (ddPCR) partitions CSF DNA into thousands of microdroplets, enabling absolute quantification and greater sensitivity than qPCR, particularly in samples with low bacillary burden [50,71]. Diagnostic performance depends on the molecular target used, with multicopy IS6110 outperforming the single copy gyrB gene. ddPCR-IS6110 achieved sensitivities ranging from 25% to 73.1% and a specificity of 97% [71], outperforming smear microscopy, culture, qPCR, and Xpert MTB/RIF, even with CSF volumes as low as 400 µL [71]. However, its clinical implementation remains limited by costs, infrastructure demands, and the need for advanced standardization.

ProbeTec ET, an isothermal strand displacement amplification assay, shows limited performance in TBM because it is not optimally designed for cerebrospinal fluid samples [55]. In clinical studies, it demonstrated high specificity (100%) but low sensitivity (50%) in CSF, performing consistently worse than Xpert MTB/RIF [55]. When evaluated against CRS or culture in extrapulmonary TB, ProbeTec ET achieved sensitivities ranging from 72.9% to 74.4%, which remained lower than those reported for Xpert MTB/RIF [55]. Therefore, despite its high specificity, the clinical role of ProbeTec ET in TBM remains secondary and complementary.

Reverse hybridization assays, such as GenoType MTBDRplus, enable simultaneous detection of *M. tuberculosis* and resistance to isoniazid and rifampicin through multiplex PCR and probe hybridization [47]. Their performance is limited in paucibacillary CSF and requires specialized infrastructure and standardized DNA extraction. In people living with HIV with suspected TBM, MTBDRplus achieved a sensitivity of 76.3%, outperforming culture (65.7%) and Xpert MTB/RIF (63.2%) but remaining inferior to qPCR (81.1%) [47]. Accordingly, MTBDRplus is recommended as a complementary NAAT when Xpert MTB/RIF is negative and clinical suspicion persists, providing additional resistance information in specialized settings.

Metagenomic next-generation sequencing (mNGS) enables untargeted detection of *M. tuberculosis* and alternative pathogens in CSF without prior pathogen selection, broadening etiological assessment in paucibacillary central nervous system infections [72,73]. Across clinical studies, mNGS achieved sensitivities of 70–85% in definite TBM and 7–63% in probable or possible cases, with specificity close to 100%, consistently outperforming Xpert MTB/RIF, conventional PCR, and culture [68,74,75]. A meta-analysis reported pooled sensitivities of 61–62% and specificities ≥ 98%, confirming robust diagnostic performance even in low-bacillary-load samples [72]. Diagnostic yield improves in inflammatory CSF and when combined with biomarkers such as hyperproteinorrachia or pleocytosis, reaching accuracy > 90% [76]. However, high costs, bioinformatic requirements, and contamination control limit routine clinical implementation, confining mNGS to specialized or referral settings.

Nanopore sequencing platforms, such as Oxford Nanopore Technologies, provide real-time, long-read sequencing that enables direct identification and genomic characterization of *M. tuberculosis* from low-volume CSF samples [77]. In a cohort evaluated using uniform case definitions, Nanopore sequencing achieved sensitivity of 77.8% and specificity of 100%, markedly outperforming culture (11.1%) and Xpert MTB/RIF (13.3%) [77]. Despite advantages including portability and rapid turnaround, lower base-calling accuracy compared with short-read sequencing requires additional error-correction algorithms and may limit detection of clinically relevant variants in low-DNA-burden contexts. Consequently, Nanopore sequencing currently serves as a high-potential but specialized diagnostic tool, primarily applicable in advanced or research settings.

In summary, complementary molecular assays expand diagnostic capacity beyond Xpert MTB/RIF and Ultra, particularly in paucibacillary CSF or when first-line NAAT yield negative results. Evidence indicates that multitarget LAMP and ddPCR provide the greatest incremental gains in sensitivity, whereas qPCR and MTBDRplus add value within sequential diagnostic algorithms in selected high-suspicion settings, while ProbeTec ET offers limited benefit in CSF samples. Collectively, these findings support a stepwise molecular diagnostic framework in which negative initial NAAT results do not exclude TBM. However, variability in sensitivity, technical complexity, and incomplete laboratory standardization continue to limit widespread clinical implementation and underscore the need for integrated diagnostic strategies tailored to resource availability and clinical context.

**Table 4:** Molecular diagnostic assays for tuberculous meningitis and their role in clinical diagnostic algorithms.

Test Characteristics and Performance	Advantages/ Disadvantages	Level of Recommendation	Key Observations	References
Xpert MTB/RIF Cartridge-based PCR Target: *rpoB* Sens: 13–100% Spec: (≥98%).	Advantages: Automated, rapid (~2 h), detects rifampicin (RIF) resistance; high specificity. Disadvantages: Highly variable sensitivity in probable/possible TBM; requires basic infrastructure and continuous power supply; cartridge cost.	First-line test (WHO/IDSA) as the initial NAAT for TBM; a negative result does not exclude TBM.	Sensitivity depends on CSF volume (≥3–5 mL increases yield; <2 mL reduces performance). Better performance in definite cases; a negative result does not exclude TBM. Lower sensitivity in probable/possible cases.	[40,47,51,53,54,56,57,59,60,64,69]
Xpert MTB/RIF Ultra Multicopy PCR Target: *IS6110/IS1081* Sens: ~35–88% Spec: 91–100%	Advantages: Higher sensitivity in paucibacillary settings; useful in pediatric populations; high specificity; detects RIF resistance. Disadvantages: Reduced sensitivity in “possible” TBM categories; trace results require careful interpretation; same platform and consumable requirements as Xpert MTB/RIF.	Preferred initial test for TBM across all groups (including PLWH and children); recommended by WHO as the NAAT of choice.	Higher sensitivity in paucibacillary samples. Requires ≥ 2 mL CSF for optimal performance. Specificity is more variable than Xpert MTB/RIF.	[56,58,61,62,63,64]
TrueNat MTB/MTB Plus Portable PCR Target: *nrdB* Sens: 83.75% Espc: 88.75%	Advantages: Portable; low infrastructure requirements. Disadvantages: Limited evidence in CSF; lack of robust validation against culture or composite reference standards (CRS).	Under evaluation; potential alternative in resource-limited settings where Xpert/Ultra are unavailable; does not replace first-line NAAT.	Performance comparable to Xpert MTB/RIF, but inferior to Ultra. WHO does not recommend routine CSF use due to limited validation. Useful as a portable alternative.	[25,70]
Alerta MTB qPCR (MTB Q-PCR) Multicopy insertion sequence Target: *IS6110* Sens: ~81% Spec: NR	Advantages: High sensitivity in people living with HIV; performance superior to several NAATs on the same cohort; useful when bacillary load is high. Disadvantages: Specificity not reported; evaluated only against another NAAT (MTBDRplus); limited availability; not automated.	Second-line and complementary test in PLWH, within sequential diagnostic algorithms; not intended as a standalone assay.	High sensitivity in PLWH. Recommended within sequential diagnostic algorithms.	[47]
PCR CapitalBio (RT-PCR) Conventional PCR Target: *IS6110* Sens: 48% Spec: 100%	Advantages: Useful for TBM confirmation (especially in PLWH); short turnaround time (~3 h); technology available in many reference laboratories. Disadvantages: Intermediate sensitivity, particularly in paucibacillary cases; requires molecular infrastructure and trained personnel; slower than cartridge-based assays	Second-line/confirmatory test when Xpert/Ultra are unavailable or when additional testing is required.	Suitable as a confirmatory assay. Requires ≥2 mL CSF.	[69]
ddPCR (*IS6110*-ddPCR) Digital PCR Target: *IS6110–gyrB* Sens: 25–73% Espc: 97%	Advantages: Very high analytical sensitivity compared with conventional qPCR; high specificity in paucibacillary settings. Disadvantages: Costly and complex; requires specialized equipment and basic bioinformatics; limited availability.	Advanced second-line test. Use in reference centers when automated NAATs are negative and clinical suspicion persists.	High analytical sensitivity in CSF with low bacillary content. Performance reported with very low CSF volumes (~0.4 mL).	[71]
LAMP (TB-LAMP) Amplificación isotérmica Target: *IS6110*, *sdaA* Sens: ~53–70% Spec: 66–78%	Advantages: Rapid technique (<1 h); does not require a thermocycler; feasible in resource-limited settings. Disadvantages: Lower specificity compared with Xpert; higher risk of post-amplification contamination; performance highly dependent on reference standard and CSF volume.	Alternative in very resource-limited settings, such as remote or rural areas when cartridge-based NAATs are unavailable.	Variable specificity. Useful in low-resource settings. Requires strict standardization. Not suitable as a first-line diagnostic test.	[47,67]
MLAMP multitarget Isothermal amplification Target: *IS6110*, *IS1081*, *sdaA* Sens: ≥88% Spec: 100%	Advantages: Sensitivity comparable to Xpert Ultra in some series; useful in PLWH (~80%); short processing time. Disadvantages: Does not detect drug resistance; limited evidence; risk of contamination.	Promising/under evaluation as a high-performance tool in resource-limited settings; additional validation required.	Multitarget design improves performance in paucibacillary CSF. High accuracy compared with composite reference standards and culture. Effective with limited CSF volumes (~1–2 mL).	[63]
GenoType MTBDRplus Reverse hybridization (line probe assay) Target: *rpoB*, *katG*, *inhA* Sens: 76% (PLWH with suspected TBM) Spec: NR	Advantages: Detects MTB and resistance mutations to rifampicin (RIF) and isoniazid (INH). Disadvantages: Requires specialized infrastructure and trained personnel; turnaround time 2–8 h; limited sensitivity in CSF with low bacillary load.	Complementary test when Xpert/Ultra are negative and clinical suspicion persists.	Provides additional resistance information. PLWH were not included in some studies. Use restricted to specialized centers.	[47]
ProbeTec ET (SDA) Strand displacement amplification Target: *IS6110.* Sens: 50% Spec: 100%	Advantages: High specificity in CSF; short analysis time (<3 h); lower technical complexity than conventional PCR. Disadvantages: Low sensitivity in CSF; not optimized for CSF samples; inferior performance compared with automated NAATs.	Restricted use/legacy platform in laboratories with existing infrastructure; not recommended as a new investment.	Designed for respiratory samples. Limited performance in TBM despite high specificity.	[55]
Metagenomic next-generation sequencing (mNGS) Short-read sequencing Sens: 61–62% Definitive: 70–85%. Probable/possible: 7–63% Spec: ≥98%	Advantages: Broad “pan-pathogen” detection; identifies *M. tuberculosis* and alternative pathogens; high specificity in paucibacillary CSF. Disadvantages: High cost; requires sequencing platforms, bioinformatics expertise, and strict contamination control.	Advanced/tertiary diagnostic tool mainly for research or specialized reference centers.	Improved performance in inflammatory CSF or in the presence of host-response biomarkers. Useful when the etiological diagnosis remains uncertain.	[68,72,73,74,75]
ONT Real-time long-read sequencing Sens: ~78% Spec: 100%	Advantages: Real-time results (<6 h); increased specificity; enables strain typing and resistance profiling; higher sensitivity than culture and Xpert in some series. Disadvantages: Platform still under development; limited standardization; requires specialized infrastructure and expertise; high cost.	Advanced/research-based tool. Use restricted to specialized or research centers.	Diagnostic promise. Evidence still limited to selected cohorts.	[77]

Note: This table provides a comparative overview of molecular assays evaluated for the diagnosis of TBM, organized according to their role within clinical diagnostic algorithms. The ordering of the assays reflects a combination of their level of support in international guidelines and expert consensus, when available, as well as operational feasibility, degree of standardization, technical complexity, turnaround time, and diagnostic performance reported in CSF. Assays categorized as first-line correspond to NAAT that are widely validated and recommended by international guidelines as initial diagnostic tools. Second-line or complementary assays include methods with lower standardization, more variable diagnostic performance, or greater technical complexity, which may help clarify the diagnosis when clinical suspicion remains high and first-line tests are negative. Finally, advanced or tertiary techniques are primarily grouped based on their methodological complexity, such as mNGS or ONT, whose use is generally restricted to specialized centers. The observations highlight relevant operational and clinical considerations, including dependence on sample volume, performance in paucibacillary settings, utility in PLWH, and the ability to detect resistance to RIF and INH, when applicable. A negative molecular result does not exclude TBM and should be interpreted in an integrated manner alongside clinical findings, CSF parameters, and other microbiological tests. NR, not reported.

### 3.3. Clinical Integration of Molecular Assays and Diagnostic Perspectives

Advanced molecular assays represent the highest level of diagnostic complexity in TBM and encompass technologies such as LAMP/MLAMP, hybridization or SDA-based assays (GenoType, ProbeTec), and sequencing platforms (mNGS, Nanopore). These tools provide rapid results, genomic characterization, and simultaneous detection of resistance, offering valuable diagnostic support in scenarios where etiological confirmation is challenging.

However, clinical applicability remains constrained by important limitations, including interlaboratory variability, high costs, specialized infrastructure requirements, and lack of standardization across multiple analytical steps. In this regard, the WHO (2025) positions these assays as complementary or advanced research tools, particularly useful when clinical suspicion remains high and automated NAAT yield negative results [23]. Collectively, these technologies support a transition toward a hybrid diagnostic approach in which immunological biomarkers, molecular assays, and genomic analyses enhance diagnostic sensitivity without compromising specificity.

In continuity with this evolution, the available evidence demonstrates a substantial expansion of the diagnostic armamentarium; however, the real-world performance of each technique depends on clinical and operational variables that can significantly modify diagnostic yield. Factors such as bacillary load, CSF volume and handling, HIV coinfection, and laboratory technical capacity directly influenced sensitivity and specificity. These sources of variability partly explain the heterogeneity observed across studies and justify the need for stepwise diagnostic algorithms that integrate microbiological, molecular, and immunological methods to maximize diagnostic confirmation, particularly in paucibacillary cases or when initial tests are negative.

Comparative analyses indicate that Xpert MTB/RIF Ultra maintains the most favorable balance between sensitivity, specificity, turnaround time, and operational feasibility, and therefore continues to serve as the international diagnostic reference. Nevertheless, its reduced sensitivity in “possible” categories and its dependence on adequate CSF processing underscore the importance of incorporating complementary assays within sequential algorithms to avoid false-negative results. Multilevel integration that combines automated NAAT, complementary molecular tests, and sequencing technologies offers a flexible framework capable of adapting to different levels of healthcare complexity. This model supports more timely therapeutic decisions aligned with the epidemiological and operational realities of each setting.

## 4. Conclusions

The synthesized evidence demonstrates substantial progress in the diagnosis of TBM. The combination of clinical and radiological criteria, CSF paraclinical analysis, and molecular assays of varying complexity improves etiological confirmation and shortens critical time to therapeutic decision-making. Although culture retains an indispensable confirmatory role and enables drug susceptibility testing, its prolonged turnaround time necessitates reliance on available molecular findings for initial treatment decisions.

Automated NAAT have become firmly established as first-line diagnostic tools. Xpert MTB/RIF Ultra remains the preferred initial assay, while platforms such as TrueNat and LAMP expand access in peripheral settings or environments with limited infrastructure. In complex or paucibacillary cases, or when drug resistance is suspected, platforms such as ddPCR and mNGS provide incremental diagnostic value. Importantly, diagnostic performance does not depend solely on the platform used, but also on CSF volume and handling, preanalytical standardization, and appropriate selection of molecular targets.

The future of TBM diagnosis is oriented toward integrated algorithms that combine biomarkers, high-sensitivity NAAT, and advanced neuroimaging. This technological convergence is expected to enhance overall sensitivity without compromising specificity and to facilitate a more individualized diagnostic approach. Nonetheless, fundamental challenges related to standardization, cost, accessibility, and availability of trained personnel persist. Addressing these gaps will require implementation of tiered diagnostic models tailored to levels of care, reinforcement of technical training, and establishment of robust quality-control mechanisms. Only through sustainable and equitable operational strategies can the full impact of these technologies be consolidated in routine clinical practice.

## Data Availability

No new data were created or analyzed in this study. Data sharing is not applicable to this article.
